# No Time to Lose: Cases of Anticoagulant Reversal Before Thrombolysis in Acute Ischemic Stroke Patients

**DOI:** 10.7759/cureus.21406

**Published:** 2022-01-19

**Authors:** Sheyar Amin, Karl A Kasischke, Kareem Elsayed, W. Scott Burgin, David Z Rose

**Affiliations:** 1 Neurology, University of South Florida Morsani College of Medicine, Tampa, USA

**Keywords:** anticoagulation reversal, thrombolytic, ischemic stroke, anticoagulation therapy, atrial fibrillation

## Abstract

Direct oral anticoagulant (DOAC) reversal before intravenous thrombolysis (IVT) in acute ischemic stroke (AIS) patients is well-documented in Europe, specifically for dabigatran: the selective humanized monoclonal antibody fragment idarucizumab, given to neutralize dabigatran prior to IVT, was associated with improved outcomes post-IVT. However, in the United States, this approach is rarely reported and not endorsed by guidelines. Therefore, further reporting on this is needed and neuroradiographic correlation may help validate this concept. At our hospital in Tampa, Florida, two octogenarians with atrial fibrillation, adherent with the DOAC dabigatran, presented with AIS shortly after symptom onset. Both received idarucizumab, then IVT. Clinical outcomes, treatment times, and perfusion-based neuroradiographic parameters were assessed. Patient A had a 41 ml penumbra on computed tomography perfusion (CTP) scan that decreased to 15 ml in final infarct volume on follow-up imaging, resulting in a 26 ml penumbral salvage (63.4%), and National Institutes of Health Stroke Scale (NIHSS) improved from 11 to 9 . Patient B had a 23 ml penumbra on CTP that decreased to 0.5 ml on follow-up imaging, resulting in a 22.5 ml penumbral salvage (97.8%), and NIHSS improved from 9 to 4. Neither developed bleeding complications. Both had delayed door-to-needle times but nevertheless demonstrated clinical neurological improvements.

In our limited experience, IVT after immediate DOAC reversal in AIS patients on dabigatran was associated with clinical improvement in NIHSS by 2 to 5 points (with no neuroworsening), and penumbral salvage of ischemic brain tissue on neuroimaging ranging from 63.4% to 97.8%. Further reporting on this may lead to greater IVT use and better outcomes in “DOAC failures”, especially for patients without other acute treatment options such as mechanical thrombectomy. Research into other anticoagulant reversal agents pre-IVT in AIS is also warranted.

## Introduction

Despite the clinical effectiveness of direct oral anticoagulants (DOAC) for patients with atrial fibrillation (AF), an acute ischemic stroke (AIS) may occur while anticoagulated anyway; this breakthrough event is given the general term, “DOAC failure.” In four landmark randomized trials, the “DOAC failure” rate was 1.1% for dabigatran, 1.7% for rivaroxaban, 1.3% for apixaban, and 1.2% for edoxaban [1-4]. In an analysis of seven prospective cohort studies, the “DOAC failure” rate was somewhat higher, at 4.4% per year [5]. Many possible reasons exist for “DOAC failure”-whether related to the pharmacokinetics or genomics of the medication itself (i.e., decreased DOAC absorption, clearance, or metabolism) or related to an error by the user or prescriber (i.e., incorrect DOAC dose or frequency, noncompliance, etc.) [6] Another reason for “DOAC failure” may be alternative stroke etiologies (other than AF) in which DOAC therapy is not indicated (i.e., lacunar disease, arterial stenosis, vasculitis, hypercoagulability/malignancy, etc.) [6]. Because these scenarios are not uncommon, the question is: what if an AF patient arrives at an emergency room with an AIS “within the window” for intravenous thrombolysis (IVT) but was adherent to their DOAC recently? The latest American Heart/Stroke Association (AHA/ASA) guidelines on this topic do not recommend IVT for patients who have taken their last DOAC within 48 hours (class III: Harm; level of evidence C-EO: consensus expert opinion) [7]. Although anticoagulant reversal agents are widely available in the United States, administered for bleeding and other indications, their use in anticoagulated patients with AIS, followed by IVT, is rarely reported.

In Europe, however, multiple centers have reported successful administration of IVT after reversing the DOAC dabigatran with the selective humanized monoclonal antibody fragment idarucizumab (Praxbind™, Boehringer-Ingelheim, Rhein, Germany) [8]. A collective, retrospective analysis in 2020 [8] found 78% of AIS patients (n=80) on dabigatran who received IVT after idarucizumab improved 7 points in their National Institutes of Health Stroke Scale (NIHSS). Similarly, a 2021 systematic literature review [9] of AIS patients (n=251) showed a statistically significant 6-point NIHSS improvement, with rates of hemorrhagic transformation, symptomatic intracranial hemorrhage, and mortality comparable to previous IVT studies on non-anticoagulated patients. Therefore, unlike the AHA/ASA guidelines, the 2021 European Stroke Organisation (ESO) guidelines [10] state that for dabigatran users, IVT after reversal with idarucizumab is suggested over no IVT.

Few reports of this reversal-then-thrombolytic approach in the United States exist: a 69-year-old northern California man had an 11-point NIHSS improvement, and a 73-year-old southern California man improved 20 points [11,12]. At our Comprehensive Stroke Center in Tampa, Florida, two right-handed octogenarians presented about a month apart with acute left hemiparesis within an IVT treatment window but with the contraindication of recent dabigatran use. We discussed with these patients and their families regarding both the AHA/ASA guidelines and the European experience, the lack of controlled trial data for the use of idarucizumab in this setting, and reviewed the risks and benefits of conventional IVT. They expressed understanding and provided informed consent for treatment. Unique to our reporting of this approach, we also performed pre- and post-treatment volumetric analyses of ischemic versus infarcted brain tissue.

## Case presentation

Patient A

**Figure 1:**
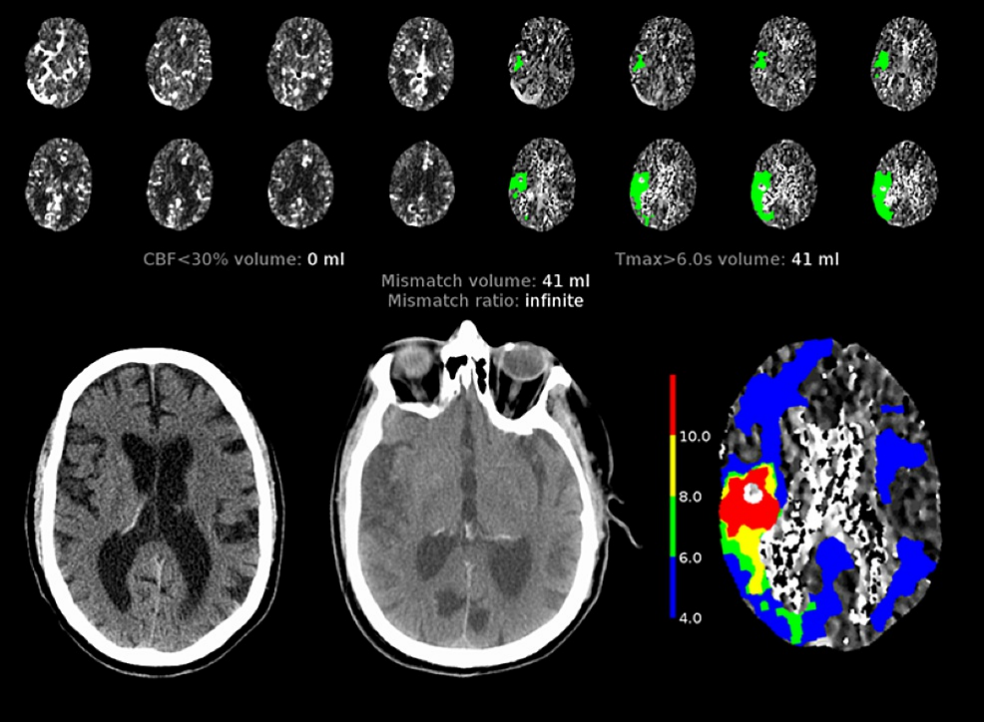
Scans of Patient A Patient A’s initial CT perfusion scan (top row) showed a moderate-sized 41 ml penumbra in a right MCA branch territory. The infarcted area is not visualized on the initial CT head (bottom row, left) while the final infarct in the right temporal region can be seen in the final CT scan (bottom row, middle). The final infarct volume corresponds to the Tmax > 10.0 s (bottom row, right) of 15 ml.

An 83-year-old right-handed man with AF, hypertension, ischemic cardiomyopathy, and a defibrillator, presented 50 minutes after the acute onset of the subjective left arm and leg “heaviness” while driving. Two weeks prior, he developed dark stools after starting colchicine for gout. His primary care doctor held his longstanding dabigatran for 7 days, restarting 2 days before the AIS presentation (last DOAC dose was 3 hours before symptom onset). NIHSS was 11, non-contrast computed tomography (CT) was unremarkable, and CT-angiogram (CTA) and CT-perfusion (CTP) revealed a distal right M2 middle cerebral artery (MCA) occlusion with a corresponding 41 ml perfusion mismatch, defined as Tmax > 6.0 s volume (Figure 1, RapidAI, CA).

After conversations with the patient and his family, and then with pharmacy, idarucizumab was given, followed by a 15-minute, pharmacy-requested treatment delay before IVT initiation, based on a previously published case report [12]. Total elapsed time, including discussions, reversal, treatment delay, and the IVT bolus, was 3 hours from time last known normal, with a door-to-needle (DTN) time of 133 minutes. Our neurointerventional team initially felt that the clot was too distal in the M2 branch but later performed a mechanical thrombectomy (MT) with partial recanalization (at 342 minutes from onset).

The 24-hour CT revealed a smaller final stroke volume (15 ml) than expected based on CTP. The measured penumbral salvage was 26 ml (63.4%). No overt bleeding occurred. His defibrillator precluded MRI. An alternate DOAC, apixaban, was started 2 days later. Discharge NIHSS was 9, and modified Rankin Score (mRS) was 4, but he was lost to follow-up.

Patient B

**Figure 2:**
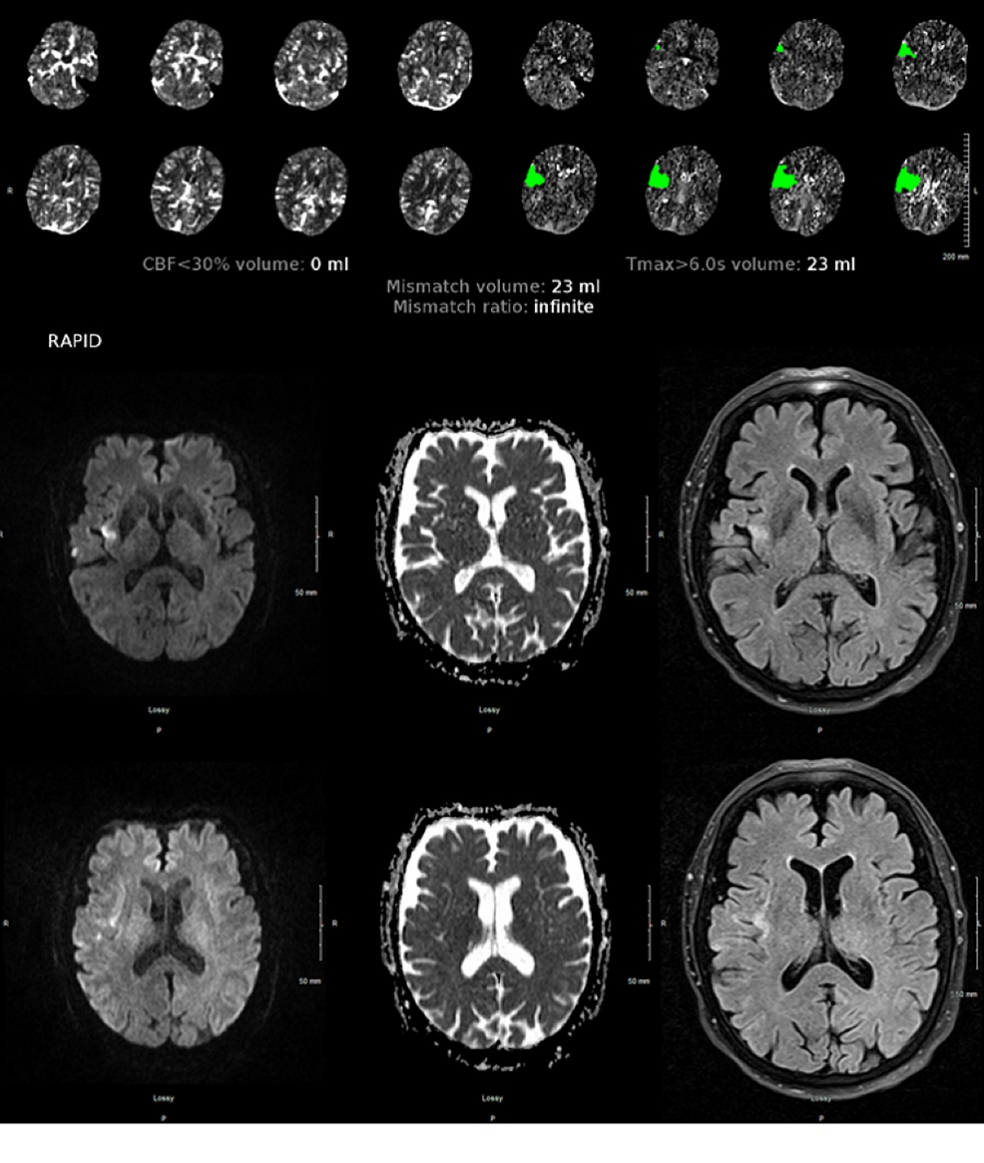
Scans of Patient B Patient B’s initial CT perfusion scan (above) showed a moderate-sized 23 ml penumbra in a right MCA branch territory. However, MRI brain (below) revealed a final infarct volume of only 0.5 ml.

An 84-year-old right-handed woman with AF and hypertension presented 30 minutes after the sudden onset of left face, arm, and leg weakness. Her last dabigatran intake was that morning. NIHSS was 9, CT and CTA were unremarkable, and CTP showed a 23 ml mismatch in the right insular region (Figure 2). After conversations with the patient and her family, she received idarucizumab followed by immediate IVT (based on feedback from the first case, the pharmacy did not request any treatment delay). DTN was 122 minutes. MRI showed a small stroke in the right insula (volume of 0.5 ml determined by computer-aided three-dimensional volumetric analysis of diffusion restriction), suggesting penumbra salvage of 22.5 ml (97.8%). Discharge NIHSS was 4 and mRS was 2. Dabigatran was restarted in 4 days. For over a year, she has followed up with neurovascular clinic and cardiology clinic, and has continued to demonstrate neurologic improvement, and recently had left atrial appendage closure to avoid long-term use of anticoagulation.

 

## Discussion

Administration of IVT after reversal of the DOAC dabigatran is well-established in Europe, with a panoply of data showing significant improvements in clinical outcomes for AIS patients [8,9]. This logical approach is supported by updated ESO guidelines [10]. However, despite the availability and use of idarucizumab in United States hospitals to reverse dabigatran for bleeding and emergency surgery/procedure since 2015, the latest AHA/ASA guidelines have not been amended to recommend this strategy [7]. Besides the potential clinical benefit in NIHSS seen in the multiple reports from Germany and the two patients from California [8,9,11,12], our cases also uniquely demonstrate the potential neuroradiographic benefit of IVT, post-reversal of dabigatran by idarucizumab. Both of our patients had similar demographics: age (octogenarians), handedness (right), stroke location (distal branch right MCA territory syndromes), NIHSS scores (9-11), amount of at-risk brain tissue ischemia (23-41 ml), and very recent DOAC use (a few hours prior to symptom onset). Also, both patients presented within the 3-hour window, and after conversations and informed consent (with alert patient and family present), both received idarucizumab followed by IVT. NIHSS improved in both patients, and neither exhibited a hemorrhagic complication. These clinical outcomes parallel to the existing literature for IVT post-idarucizumab outcomes as well as outcomes in non-anticoagulated patients [8,9]. Perhaps ideal candidates for this approach will have similar clinical characteristics-older age, on dabigatran, “in the window,” with an acute, medium-sized, partial cortical ischemic stroke that is suboptimal for thrombectomy (too distal) and yet large enough (clinically and radiographically) to consider the thrombolysis benefit worth the risk.

Unique to our report, both patients experienced volumetric penumbral salvage: 26 ml for Patient A (from 41 to 15 ml) and 22.5 ml for Patient B (from 23 to 0.5 ml). In hindsight, penumbral salvage for Patient A may have been even greater with an earlier MT with better recanalization, or if the pharmacy had not requested the 15-minute treatment delay. Pharmacy rescinded any treatment delay for Patient B (who had slightly better DTN) after feedback regarding the onset-of-action of idarucizumab: within seconds of a standard 5-g dose, the biological activity of circulating dabigatran molecules is reversed due to immediate binding with monoclonal antibody fragments [13,14].

In clinical practice, so-called “DOAC failures” are most commonly related to noncompliance or dose/frequency error rather than dysfunction in DOAC pharmacokinetics or genomics (such as decreased DOAC absorption, clearance, or metabolism). Regardless, “DOAC failures” do occur in about 1-5% of cases [1-5], many of whom will be “in the window” for IVT. Patient A, for example, was instructed to hold dabigatran because of possible melena related to colchicine. After a reasonable workup, his gastroenterologist could not identify a bleeding source and therefore had authorized resuming the DOAC after a week off of it. During the time while unanticoagulated, a clot may have formed in Patient A’s left atrial appendage, and embolized to the brain shortly after the DOAC was restarted. Patient B also presented “in the window” for IVT while anticoagulated, and it was confirmed she had very recent and consistent intake of dabigatran. Therefore, based on our understanding of the literature, and after a case-by-case review of the scans, vitals, clinical history, and exam in the emergency room, both patients received idarucizumab and then IVT. Fortunately, both likely fared no worse than without IVT, and both actually improved both clinically and, unique to our report, neuroradiographically.

The AHA/ASA guidelines mention that IVT can be considered if the last known DOAC intake was >48 hours before AIS onset, or if sensitive laboratory tests such as thrombin time and anti-Xa assays are normal. Unfortunately, these assays are not universally (or rapidly) available at many centers [15], and may considerably delay DTN times. As witnessed in our cases, DTN times were already considerably delayed because of the extensive discussions with patients and families about discrepancies in the American versus European guideline recommendations.

Limitations of this report include the following: (1) Patient A underwent both IVT and MT which may have led to further clinical improvement than IVT alone, (2) different DOAC agents (dabigatran and apixaban) were started in the subacute phase after the acute strategy, and (3) at different times, which may have confounded hemorrhage risk at a follow-up visit, and (4) this report by itself on face value can only provide loose conclusions due to its size: we describe only two patients who underwent IVT after DOAC reversal. Nevertheless, data on this approach is sparse in the United States, and our findings add to its safety record of the well-documented European experience and is congruent with the ESO guideline update. Moreover, our report is the first to include brain perfusion data and volumetric analysis, strengthening the therapeutic plausibility of IVT post-reversal in patients who may not qualify for MT based on current guidelines [14] or other reasons. MT instead of IVT is an option for some anticoagulated patients with AIS, however, not all centers provide neurointerventional capabilities, coverage, or logistics, and distal emboli may not be targetable for MT. Furthermore, IVT post-reversal and MT are not necessarily mutually exclusive: an “expert opinion” article from 2017 noted: “If a delay to initiate [MT] is anticipated and the patient meets standard eligibility criteria for [IVT] apart from their dabigatran use, then [idarucizumab] should be administered, immediately followed by [IVT]” [14].

Research is sorely needed for all patients on any anticoagulant with AIS who are otherwise eligible for IVT - incorporating other reversal agents besides idarucizumab: examples include prothrombin complex concentrate (indicated for patients on warfarin with acute major bleeding or the need for urgent surgery or other invasive procedure), and andexanet-alfa (indicated for patients on apixaban and rivaroxaban with life-threatening or uncontrolled bleeding) [15]. Given the time-sensitive, hyperacute nature of an AIS, it is reasonable to consider IVT administration an urgent procedure.

Although a randomized trial of idarucizumab pre-IVT in AIS may have been useful, the growing body of reports, both large and small, make such a study (with a placebo arm) unlikely to gain approval after so many positive outcomes with this approach. If AHA/ASA guidelines were to mirror the ESO recommendations, even though based on limited data and expert opinion, then conceivably, more patients with AIS, who happen to be on dabigatran, who otherwise would not qualify for IVT, instead could now receive it. This small update could result in improved DTN times, more penumbral salvage, and ultimately, better clinical outcomes, as the literature has suggested. We have no time to lose.

## Conclusions

Clinical and neuroradiographic improvement with penumbral tissue salvage is possible with IVT after DOAC reversal in AIS patients taking dabigatran. This approach is rarely reported in the United States, but is well-established in Europe and is included in ESO guidelines. An update to the AHA/ASA guidelines that recommends this approach may lead to greater IVT use and better outcomes in “DOAC failures,” especially for patients without other acute options such as MT. Research into other anticoagulant reversal agents pre-IVT in AIS is also needed.
